# Survival-oriented personality factors are associated with various types of social support in an emergency disaster situation

**DOI:** 10.1371/journal.pone.0228875

**Published:** 2020-02-12

**Authors:** Motoaki Sugiura, Rui Nouchi, Akio Honda, Shosuke Sato, Tsuneyuki Abe, Fumihiko Imamura

**Affiliations:** 1 International Research Institute of Disaster Science, Tohoku University, Sendai, Japan; 2 Institute of Development, Aging and Cancer, Tohoku University, Sendai, Japan; 3 Faculty of Informatics, Shizuoka Institute of Science and Technology, Fukuroi, Japan; 4 Graduate School of Arts and Letters, Tohoku University, Sendai, Japan; Swinburne University of Technology Faculty of Health Arts and Design, AUSTRALIA

## Abstract

Mutual help is common in human society, particularly during a disaster. The psychological processes underlying such social support are of interest in social and evolutionary psychology, as well as in the promotion of community resilience. However, research in terms of personality factors or support types is sporadic and has yet to address actual emergency situations. In this study, we analyzed survey data from survivors of the 2011 Tohoku earthquake and tsunami. The data included five types of social support occurring during the evacuation from a potential tsunami area: providing and receiving actual help and oral encouragement, as well as perceived support. The personality factor items included the Big Five dimensions and eight “power to live” factors, which were identified as advantageous for survival during this disaster. While none of the Big Five dimensions were associated with social support, six of the power to live factors were. Altruism, problem solving, etiquette, and self-transcendence contributed to the provision of actual help. Leadership and active well-being contributed to oral encouragement with the latter contributing also to perceived support. The findings were largely consistent with the literature in a non-emergency context. The relevance of the majority of these pro-survival personality factors to social support appeared to support the view that the propensity to cooperate in service of human survival in a disaster situation is primarily a social, rather than an individual, phenomenon, and encourages research on the mechanisms underlying how personality factors provide a benefit to both the individual and their community.

## Introduction

In human society, people help each other survive harsh realities. Mutual help is particularly common during emergencies in disaster situations [1, 2] and, rather than being limited to members of an established community, can also occur among strangers. During these periods, altruistic behavior predominates over egoistic behavior, bringing a sense of happiness to damaged communities in a phenomenon known as a post-disaster utopia [3].

The psychological basis of such social support is of interest in a variety of fields. The universality of social support outside of kin relationships is unique to humans and, thus, its underlying mental machinery remains the subject of ongoing discussion from various evolutionary perspectives [4–7]. Many researchers consider this type of behavior to be an enigma that is apparently contradictory to the individual propensity toward survival and have attempted to explain it by adopting various mediating social processes such as reciprocity [7], costly signaling [6], and cultural adaptation [4]. However, other researchers have taken these behaviors for granted by assuming that human survival is primarily a social, rather than an individual, process by referencing early humans. Early human populations had to overcome hostile environments by cooperating with others within a community because the necessity for group survival took precedence over intra-specific competitions [5]. Thus, understanding the role of psychological factors has also been of practical interest with respect to interventions for enhancing individual and community resilience [8, 9]. However, to date, empirical research has yet to explain fully the relationship between individual and community survival and the provision and receipt of support have only been investigated independently [10]. In fact, empirical research has yet to address actual emergency disaster situations in which the survival of individuals as well as the community both matter.

Multiple motivational and personality factors have been shown to promote helping behaviors in psychological studies over the past 50 years, with a particular focus on whether they are altruistic or egoistic [11, 12]. Behaviors can have altruistic or other-beneficial motivations that are triggered by empathic concerns for the person being helped [13–15] or by the social norms of helping [16–19]. In contrast, such behaviors may also be based on egoistic self-beneficial motives, such as the enhancement of self-esteem [20–22], acquisition of reputation [23], acquisition of personal networks or skills [24, 25], and/or the resolution of distress caused by a specific situation [26]. Egoistic motivation is typically associated with helping in the relevant context. For example, people who are oriented toward enhancing their own self-esteem help others when their own psychological need for esteem is high [20, 27] whereas those who score high for a Machiavellian personality help in the presence of others [28] and those who are sensitive to their own distress help when there is no other means of escaping their distress [29]. However, when the cost of helping is high, these egoistic motives are undermined and the influence of altruistic personality traits on behavior becomes more prominent [28, 30].

Although the personality factors that affect the receipt of support were investigated in the 1980s, few recent studies have followed-up on these findings. It has been shown that, under stressful circumstances, people with a high sense of mastery [31] or self-esteem [32] receive more social support. Other studies have suggested that different personality characteristics are related to different types of support reception. For example, personal predisposition (e.g., self-esteem), appraisal of stress, and coping strategies are related to the receipt of emotional, tangible, and informational support, respectively [33]. Furthermore, perceived social support, which is considered to be independent of the actual support that is received [34], is related to personal characteristics that are relevant to social interactions, such as social competence [35] and extraversion [36]. Perceived social support has also been associated with hardiness [37], while some suspected that the association can be attributable to age and experience, which both lead to higher levels of hardiness as well as a richer support network [38].

Although several previous works have investigated emergency situations [39–42], these studies appear to have made limited contributions to the understanding of the psychological bases of social support during emergency situations in actual large-scale disasters. First, these previous studies assessed dyadic interactions in which only the person being helped is in trouble. These situations do not appear to be comparable to disaster situations in which support providers as well as recipients are victims and both groups appraise the stressor in a similar way [43]. Second, because these studies employed situations in which an experimenter or actor pretended to be in trouble, there are limitations regarding the perceived genuineness of the situations. For example, the degree to which the participants suspected the genuineness or falsity of the situation, and reported such suspicions during debriefing as an excuse for not having helped, may be correlated with personality factors, causing a serious data selection bias [42]. Finally, these studies only addressed the provision of support and evaluated limited sets of personality traits, such as religiosity [39], responsibility denial [40], and sex roles [41, 42].

Thus, the present study aimed to determine the personality factors associated with social support during a large-scale natural disaster to elucidate more comprehensively the psychological basis of social support. The context mattered both individual and community survival, and is thought to be more relevant to situations in which human socio-psychological characteristics have been shaped throughout the history of human evolution. We analyzed the survey data of survivors of the 2011 Tohoku (or Great East Japan) earthquake and tsunami, in which more than 15,000 people were killed by a tsunami [44]. These data include evaluations of mutual social support during evacuation at the time of the earthquake; the questionnaire items pertain to helping and encouragement as well as perceived support. The other part of the dataset concerns two sets of personality factors: one set of factors that are specifically relevant to survival and another set for the Big Five personality dimensions. The former is a comprehensive set of psychological and behavioral characteristics known as “power to live” (with disasters) that has previously been identified as advantageous for survival during a disaster and includes leadership, problem-solving, altruism, stubbornness, etiquette, emotion regulation, self-transcendence, and active well-being [45].

Accordingly, the present study proposed three primary research questions. First, in the context of disaster, this study assessed whether multiple altruistic and egoistic motivations for helping [11, 12] would be activated and whether the latter would be sensitive to the cost of helping [28, 30]. Of the survival-oriented characteristics, altruism and etiquette may be categorized as altruistic helping motivations given their relevance to a higher degree of empathic concern and norm-compliance motivation, respectively. On the other hand, leadership and active well-being appear to overlap with personality factors that have previously been implicated in egoistically motivated helping [20–25]. The latter set of factors may be less relevant to the actual provision of help than to oral encouragement because egoistic motivation is undermined by the cost of helping [28, 30], such as when there is a seemingly higher cost (i.e., a risk of self-sacrifice) of actual helping in the face of an imminent tsunami. Second, the present study evaluated whether the contributions of various personal characteristics associated with support receipt [31–33, 35–37] would be replicated in a disaster context. For example, sense of mastery [31], self-esteem [32], and various coping styles [33], which have been implicated in the actual receipt of support, and hardiness [37], which has been implicated in the perceived receipt of support, appear to overlap with active well-being; it was noteworthy if the contribution of active well-being would be still valid after controlling the effect of age, which might have explained their apparent associations [38]. Furthermore, leadership, altruism, and extraversion may also be involved given their relevance to social interactions [35, 36]. Finally, we were interested in the levels that the two sets of personality factors (i.e., the power to live and the Big Five characteristics) contribute to the various aspects of social support. Although previous findings have suggested that both sets of characteristics contribute, the view that human survival is primarily a social rather than an individual process [5] may predict that survival-oriented characteristics are more relevant.

## Materials and methods

### Ethics statement

The survey protocol was reviewed and approved by the Ethics Committee for surveys and experiments of the Graduate School of Arts and Letters, Tohoku University (2012-1019-190749).

### Participants

The original survey included 1,412 survivors of the 2011 Tohoku earthquake and tsunami and the present study analyzed the data of 959 of these survivors who reported evacuating to avoid the tsunami on 11 March, 2011. Originally, a questionnaire battery was sent to 3600 residents who were randomly sampled from the electoral registers (and, thus, were ≥ 20 years of age) of tsunami-affected districts or temporary settlements in the four most populous coastal cities of Miyagi Prefecture, where damage caused by the earthquake and tsunami was most severe (see [45] for further details of the survey). In total, 1,412 questionnaires (39%) were anonymously completed and returned by mid-January, 2014. These data have previously been used to construct a “power to live” inventory [45, 46] and to analyze the psychological factors of appropriate (i.e., immediate or voluntary) evacuation when avoiding a tsunami [47].

Of the 1,412 respondents, 959 (68%) replied ‘yes’ to the question, “Did you evacuate to avoid the tsunami when the earthquake occurred on 11 March, 2011?” Of the remaining 453 respondents, 428 replied ‘No’ and 25 did not provide a reply. Of the 959 evacuees, 8% were injured, 52% lost their entire home, 40% had partially-damaged homes, and 9% lost family members.

For reference purposes, in terms of data representativeness, comparisons of the demographic information and damage characteristics between the 959 evacuees and 428 non-evacuees are reported in the S1 File. Briefly, the evacuees had a more severe level of home damage than the non-evacuees but there were no significant differences in the other factors. This suggests that the different behaviors stemmed from the perceived likeliness of a tsunami hit, which was likely to be due to geographic factors (e.g., distance from seacoast).

### Support provided and received during the tsunami evacuation

Five questions, each requiring a yes or no answer, were included pertaining to mutual support (Table 1). These items were concerned with whether respondents helped or encouraged others during the evacuation. Items pertaining to receipt of support were concerned with whether respondents were helped or encouraged by someone else during the evacuation. One question on perceived support asked whether there was someone whom the respondent could rely on during the evacuation.

**Table 1:** Questionnaire items pertaining to social support.

Support type		Item
**Provision**	**Helped**	“Did you help someone during the evacuation?”
	**Encouraged**	“Did you encourage someone to evacuate during the evacuation?”
**Receipt**	**Helped**	“Were you helped by someone during the evacuation?”
	**Encouraged**	“Were you encouraged by someone during the evacuation?”
	**Perceived**	“Was there someone whom you could rely on?”

The social support items were presented following the question “Did you evacuate to avoid the tsunami when the earthquake occurred on March 11, 2011?”; only the respondents who replied yes to this question were required to reply to the subsequent items (with yes or no responses).

### Personality factors

The power to live questionnaire is comprised of 34 items that describe a way of thinking, daily attitudes, and/or habits; the internal consistency and concurrent validity of the questionnaire have been verified [45, 46]. Descriptions and example items for each factor are given in Table 2. The participants responded using a 6-point scale (0: *not at all*; 5: *very much*). Each factor is composed of three to five items and the sum of their scores was converted to a percentile of the total score.

**Table 2:** Eight factors of the “power to live” construct.

Factor	Description	Example item
**Leadership**	Attitude or habit of gathering and organizing people	“To resolve problems, I gather together everyone involved to discuss the matter.”
**Problem-solving**	Attitude or habit of strategically tackling problems	“When I am fretting about what I should do, I compare several alternative actions.”
**Altruism**	Personality trait that causes people to care about and help others	“When I see someone having trouble, I have to help them.”
**Stubbornness**	Personality trait, attitude, or habit of sticking to one’s desires or beliefs	“I am stubborn and always get my own way.”
**Etiquette**	Attitude or habit of conforming to social norms in daily behavior	“On a daily basis, I take the initiative in greeting family members and people living in the neighborhood.”
**Emotion regulation**	Attitude or habit of endeavoring to stay calm in difficult or strained circumstances	“During difficult times, I endeavor not to brood.”
**Self-transcendence**	Awareness of the meaning of one’s life from a spiritual perspective	“I am aware that I am alive, and have a sense of responsibility in living.”
**Active well-being**	Daily practice of maintaining or improving one’s physical, mental, and intellectual status	“In everyday life, I have habitual practices that are essential for relieving stress or giving me a change of pace.”

Each factor is composed of three to five items scored using a six-point scale (0: *not at all*;– 5: *very much*).

To assess the Big Five personality dimensions, the present study employed the Japanese version of the Ten-Item Personality Inventory (TIPI-J) for which the internal consistency and concurrent validity have been confirmed [48, 49]. One positive item and one reverse item were included for each of extraversion, agreeableness, conscientiousness, neuroticism, and openness dimensions. The participants responded using a 6-point scale (0: *not at all*; 5: *very much*). For each dimension, the reverse item score was subtracted from that of the positive item, resulting in a scoring range of –5 to +5.

### Analysis

The associations of demographic factors (i.e., sex [male or female] and age [20–29, 30–39, 40–49, 50–59, 60–69, or 70+ years]) or personality factors (from the power to live and Big Five questionnaires) with the provision or receipt of social support during the tsunami evacuation were examined. The demographic data were cross-tabulated and chi-square tests were performed. The average personality factor scores were compared between the “yes” and “no” groups using an analysis of covariance (ANCOVA) with sex and age as covariates. The significance threshold was set to p < 0.05 with correction for multiple comparisons performed using the Bonferroni method (75 tests; 15 independent × 5 dependent variables).

## Results

### Frequency of social support and the effects of demographic factors

Among the respondents who evacuated to avoid the tsunami, 21% reported that they helped, and 54% that they encouraged, someone else during the evacuation. On the other hand, 12% respondents were helped, 42% were encouraged by others, and 54% perceived themselves as recipients of social support. Females reported being helped more often and had higher levels of perceived support. The effect of age was not significant for any type of support (Table 3).

**Table 3:** Frequency of social support by demographic factors.

		Provision				Receipt					
		Helped		Encouraged		Helped		Encouraged		Perceived	
		Yes	No	Yes	No	Yes	No	Yes	No	Yes	No
	*N*	196	739	505	431	114	819	396	540	502	428
**Sex**											
Male	376	84	283	207	162	24	344	135	234	146	219
Female	575	109	452	292	268	88	470	259	300	354	204
χ^2^(1) (Cramer’s *V*)		1.61	(0.042)	1.40	(0.039)	17.84*	(0.139)	8.64	(0.097)	48.84*	(0.230)
**Age (y)**											
20–29	50	10	40	22	28	6	44	21	29	30	20
30–39	95	16	77	39	54	13	80	37	56	47	46
40–49	153	35	117	89	63	21	130	68	84	85	67
50–59	183	46	133	98	82	15	164	74	106	90	88
60–69	270	52	213	138	126	33	232	104	162	137	125
70+	201	35	155	114	77	25	164	90	98	112	77
χ^2^(5) (Cramer’s *V*)		4.67	(0.071)	11.52	(0.111)	3.33	(0.060)	4.22	(0.067)	4.65	(0.071)

Data are cross-tabulated with sex and age. The samples with missing data relevant to the analysis were excluded. Chi-square tests were performed.

*p < 0.05, corrected for multiple comparisons using the Bonferroni method (75 tests; 15 independent × 5 dependent variables). Cramer’s *V* is used as the effect size.

### Effect of personality factors

The average scores for the personality factors of the support providers and non-providers are summarized in Table 4. Among the eight power to live factors, the scores for problem-solving, altruism, etiquette, and self-transcendence were higher in those who helped someone else during the evacuation. The scores for leadership and active well-being were higher in those who encouraged others. Significant differences were not observed between the support providers and non-providers in the scores for the Big Five dimensions.

**Table 4:** Association between personality factors and social support provision.

	Actual help					Oral encouragement				
**Power to live**	Yes	No	*F*(1, 823)		Partial η^2^	Yes	No	*F*(1, 825)		Partial η^2^
*N*	172	655				442	387			
Leadership	55.4 ± 19.6	50.8 ± 18.5	8.960		0.011	54.1 ± 18.5	49.0 ± 18.7	14.567	*	0.017
Problem-solving	69.7 ± 14.9	65.0 ± 15.8	11.956	*	0.014	67.0 ± 15.7	64.4 ± 15.8	5.866		0.007
Altruism	67.1 ± 15.7	62.2 ± 15.6	13.931	*	0.017	64.2 ± 15.1	62.0 ± 16.5	4.911		0.006
Stubbornness	60.8 ± 18.4	58.9 ± 16.8	1.025		0.001	59.5 ± 17.2	58.8 ± 17.3	0.403		0.000
Etiquette	85.7 ± 12.3	81.9 ± 15.5	14.644	*	0.017	83.4 ± 15.1	81.5 ± 15.1	4.012		0.005
Emotion regulation	69.5 ± 14.4	66.1 ± 16.4	6.509		0.008	68.1 ± 16.2	65.0 ± 15.7	6.269		0.008
Self-transcendence	75.5 ± 14.1	71.0 ± 15.6	13.058	*	0.016	73.2 ± 15.4	70.3 ± 15.4	7.749		0.009
Active well-being	62.7 ± 20.5	56.9 ± 20.9	9.569		0.011	60.8 ± 20.4	54.7 ± 21.0	15.670	*	0.019
**Big Five**	Yes	No	*F*(1, 841)		Partial η^2^	Yes	No	*F*(1, 843)		Partial η^2^
*N*	176	669				452	395			
Extraversion	0.34 ± 2.18	0.05 ± 1.93	3.134		0.004	0.15 ± 1.96	0.04 ± 1.99	1.122		0.001
Agreeableness	2.15 ± 1.83	2.01 ± 1.70	1.148		0.001	2.11 ± 1.65	1.94 ± 1.81	1.505		0.002
Conscientiousness	0.66 ± 2.03	0.67 ± 1.96	0.000		0.000	0.75 ± 1.83	0.56 ± 2.10	0.854		0.001
Neuroticism	0.04 ± 1.83	0.03 ± 1.90	0.034		0.000	-0.07 ± 1.79	0.15 ± 1.97	1.977		0.002
Openness	-0.14 ± 2.12	-0.25 ± 2.05	0.163		0.000	-0.18 ± 2.03	-0.30 ± 2.08	0.729		0.001

For each type of social support provision, the sample size (*N*) and score (mean ± SD) of each personality factor (power to live and Big Five) are reported separately for the providers (i.e., yes) and non-providers (i.e., no). For each personality factor, the mean scores were compared between the groups using an analysis of covariance (ANCOVA), including the age and sex factors as covariates; the F-score and partial η^2^ (effect size) are presented. Other details are the same as for Table 3.

None of the personality factors varied significantly in the scores between the support recipients and non-recipients (Table 5). The active well-being score was higher in those who perceived themselves as recipients of social support than in those who did not (Table 6).

**Table 5:** Association between personality factors and social support receipt.

	Actual help				Oral encouragement			
**Power to live**	Yes	No	*F*(1, 824)	Partial η^2^	Yes	No	*F*(1, 825)	Partial η^2^
*N*	100	728			348	481		
Leadership	54.0 ± 19.0	51.5 ± 18.8	1.388	0.002	53.1 ± 19.5	50.8 ± 18.3	2.660	0.003
Problem-solving	65.4 ± 19.2	65.9 ± 15.3	0.025	0.000	65.6 ± 15.9	65.9 ± 15.8	0.016	0.000
Altruism	64.2 ± 15.1	63.1 ± 15.9	0.130	0.000	63.1 ± 16.1	63.3 ± 15.6	0.107	0.000
Stubbornness	58.0 ± 16.9	59.5 ± 17.4	0.140	0.000	58.9 ± 17.9	59.6 ± 16.8	0.030	0.000
Etiquette	82.5 ± 17.5	82.6 ± 14.8	1.441	0.002	83.5 ± 16.1	81.8 ± 14.3	0.612	0.001
Emotion regulation	67.3 ± 20.3	66.7 ± 15.4	0.204	0.000	66.8 ± 16.1	66.7 ± 16.0	0.034	0.000
Self-transcendence	69.8 ± 19.1	72.1 ± 14.9	3.106	0.004	71.8 ± 16.1	71.8 ± 15.0	0.069	0.000
Active well-being	56.8 ± 22.5	58.3 ± 20.7	0.018	0.000	58.4 ± 22.2	57.8 ± 19.9	0.611	0.001
**Big Five**	Yes	No	*F*(1, 840)	Partial η^2^	Yes	No	*F*(1, 843)	Partial η^2^
*N*	99	745			360	493		
Extraversion	-0.05 ± 1.75	0.12 ± 2.02	1.090	0.001	0.04 ± 1.93	0.14 ± 2.02	0.664	0.001
Agreeableness	2.23 ± 1.58	2.01 ± 1.75	1.000	0.001	2.04 ± 1.68	2.03 ± 1.77	0.016	0.000
Conscientiousness	0.73 ± 2.09	0.66 ± 1.96	0.126	0.000	0.71 ± 2.00	0.64 ± 1.95	0.123	0.000
Neuroticism	-0.07 ± 1.85	0.05 ± 1.89	0.982	0.001	-0.12 ± 1.86	0.16 ± 1.89	5.302	0.006
Openness	-0.57 ± 2.12	-0.19 ± 2.05	1.153	0.001	-0.33 ± 2.01	-0.17 ± 2.10	0.469	0.001

For each type of social support received, the same data set as in Table 4 is reported separately for the recipients (i.e., yes) and non-recipients (i.e., no).

**Table 6:** Association between personality factors and perceived social support.

	Perceived support				
**Power to live**	Yes	No	*F*(1, 822)		Partial η^2^
*N*	444	382			
Leadership	53.3 ± 18.9	50.2 ± 18.6	5.125		0.006
Problem-solving	66.4 ± 16.7	65.1 ± 14.6	2.131		0.003
Altruism	63.9 ± 15.6	62.5 ± 15.9	0.609		0.001
Stubbornness	58.6 ± 17.6	60.3 ± 16.7	0.379		0.000
Etiquette	84.3 ± 14.2	80.6 ± 15.7	2.577		0.003
Emotion regulation	67.4 ± 16.9	66.1 ± 15.1	2.081		0.003
Self-transcendence	72.3 ± 15.9	71.5 ± 14.7	0.011		0.000
Active well-being	60.0 ± 20.4	56.0 ± 21.3	15.633	*	0.019
**Big Five**	Yes	No	*F*(1, 838)		Partial η^2^
*N*	450	392			
Extraversion	0.02 ± 1.95	0.21 ± 2.00	3.137		0.004
Agreeableness	2.16 ± 1.66	1.90 ± 1.80	3.307		0.004
Conscientiousness	0.64 ± 2.01	0.68 ± 1.92	0.085		0.000
Neuroticism	0.11 ± 1.81	-0.05 ± 1.97	0.310		0.000
Openness	-0.35 ± 2.06	-0.10 ± 2.05	0.406		0.000

For the perception of social support received, the same data set as in Table 4 is reported separately for the perceivers (i.e., yes) and non-perceivers (i.e., no).

## Discussion

The present study was the first to examine social support-related personality factors in the context of an actual emergency situation during a disaster. This study addressed both support provision and receipt and also evaluated various personality factors, including narrow pro-survival characteristics and broad general dimensions. The present results demonstrated associations of the various personality factors with different types of support during evacuation from the tsunami. First, altruism, etiquette, problem-solving, and self-transcendence were associated with actual helping while leadership and active well-being were associated with oral encouragement. These findings indicate that both altruistic and egoistic motivations were involved in support provision, as previously described [11, 12], also in a disaster context. Second, active well-being was associated with perceived support, which appears to be consistent with the reported contributions of hardiness to perceived support [37]. Finally, the present study found that various pro-survival personality factors, but none of the Big Five dimensions, were involved in social support. This finding is in line with the notion that individual survival and community survival are tightly connected and may suggest that the propensity to cooperate in service of human survival is primarily a social, rather than an individual, process [5].

The present findings appear partially consistent with the notion that egoistic motivation is undermined by the cost of helping [28, 30]. As expected, altruism and etiquette, which may be associated with a higher degree of empathic concern and norm-compliance motivation, respectively, were associated with actual helping. In the context of an imminent tsunami hit, this behavior leads people to risk their own life by spending substantial lengths of time with others in need of help. On the other hand, the two egoistic factors of leadership and active well-being were only associated with oral encouragement, which facilitates the evacuation of a large number of people with little time cost to oneself.

However, the associations of problem-solving and self-transcendent factors with actual helping do not appear to fit this altruism-egotism dichotomy in terms of cost sensitivity. Although the association of the problem-solving factor appears unintuitive, it makes sense when one considers that esteem-orientated personality has previously been linked with helping behavior [21, 27, 50]. For example, esteem-oriented individuals are achievement-oriented and dominant [21] and their helping behaviors are self-initiated [50] and enhanced by their awareness of competence rather than status [27], which appears to be consistent with the problem-solving item, “To resolve a problem, I first of all initiate action.” They are also less anxious [21], which is congruent with the problem-solving-related item, “The more agitated the people around me become, the calmer I become.” Although this suggests that help from a problem-solving individual is not purely altruistic (i.e., it is an esteem- or achievement-oriented behavior), it is still distinct from a purely egoistic motivation in that problem-solving was associated with costly, actual help, but not with oral encouragement.

The self-transcendence factor of the power to live construct complicates the altruism-egotism dichotomy itself. Self-transcendence apparently has two aspects. The first is a transpersonal perception of the self that extends beyond the individual, as represented by the item: “I think that my actions toward others will eventually come back to me.” Some researchers have perceived this aspect as being less egoistic and place it at the opposite end of the spectrum from self-conscious experience and self-differentiation [51] whereas others have associated transpersonal perception (i.e., the sense of oneness with another, as expressed in the tendency to help) with an egoistic motivation [52]. Another component of self-transcendence is the perception of social obligation, as represented by the item: “I am aware of the role I should play in society.”. It is plausible to associate self-transcendence with the social or personal norm of helping [16, 17, 19], which is typically studied in isolation from altruism-egotism in the field of prosocial behavior [11, 12].

The observed roles of leadership and active well-being are consistent with several lines of research regarding egoistic helping motivation. For example, these two characteristics overlap with well-accepted egoistic motivations such as developing a personal network and learning skills [24, 25], respectively. In addition to these characteristics, helping behavior may also be driven by motivations regarding one’s own self-esteem [20–22], reputation [23], and resolution of distress caused by the situation [26], which are known as egoistic helping motivations.

The present findings also suggest that personality factors influence perceived support in an emergency situation during a disaster. The observed association between active well-being and perceived support appears to be consistent with previously reported associations between emotional management ability [36], positive mood [35], and hardiness [37] and perceived support. Active well-being seems to overlap with the two former characteristics in terms of the daily practice of maintaining one’s mental health. Hardiness, which includes the commitment to daily life activities and the anticipation of change as an exciting challenge to further development [53], appears to overlap with the physical and intellectual aspects of active well-being. The claim that the association may be attributable to the effect of age [38] was not supported since the current results were obtained after controlling the effect. Nonetheless, it is still possible that these characteristics and the seeking of social support within a problem-focused coping style [38] may explain this association.

Based on the contributions of leadership and active well-being to oral encouragement and the latter’s association with perceived support, these characteristics may be key personality factors that balance individual and community survival. For example, nearby residents played a major role in encouraging people to evacuate during the 2011 Tohoku earthquake and tsunami and their role in the evacuation has been officially enshrined by a community disaster management body [54]. Interestingly, at the same time, these two characteristics simultaneously promote individuals’ immediate voluntary evacuation behaviors to avoid a tsunami when an earthquake has occurred [47]. The balance between individual and community survival may also be relevant to perceived support because this perception is likely to be shared among community members. Furthermore, leadership and active well-being contribute to the mental health of survivors in the long term [45]. It is possible that this balance stems from the characteristics related to egoistic helping motivations (i.e., for one’s own self-esteem [20–22], reputation [23], and resolution of distress caused by the situation [26]) or those related to perceived support (emotional management ability [36], positive mood [35], hardiness [37], and a problem-focused coping style [38]). Thus, these characteristics may be good targets for socio-educational interventions that promote community resilience [8]. One advantage associated with the implementation of leadership and active well-being as intervention targets is their egoistic bent in that people are happy to comply with socio-educational measures that enhance these attributes.

Although various power to live factors were associated with social support, none of the Big Five dimensions showed any such association, suggesting greater utility of the former versus the latter in predicting social support in a disaster context. Because the power to live factors are focused on survival in adversity, the finding is in line with the suggested advantage of highly specific, narrow traits over the general Big Five dimensions for predicting various socially significant behaviors [55–57]. However, this advantage may be limited to the emergency context, given that the Big Five dimensions were associated with support provision [58] or perceived support [36] in non-emergency contexts. A potential limitation of the present data concerns the use of the very short version of the Big Five inventory [48]; however, the validity of the Japanese version is high [49].

The observed associations of pro-survival personality factors with social support is consistent with the view that the propensity to cooperate in service of human survival is primarily a social, rather than an individual, process. The associations of six of the eight power to live factors with social support exceeded our expectations, given that no particular emphasis was placed on the group dynamic during the interviews for the inventory construction [45]. This finding can be understood in terms of early humans. For example, because humans rely on a community for survival, it is unlikely that any pro-survival personality factor would be independent of group survival [5]. The relevance of these pro-survival factors to aid provision was thus clear in the natural disaster context of this study. Although a variety of pro-social personality factors have been described [12], recent studies have largely focused on how they fit with the view that human survival is primarily individual, including the altruism-egoism dichotomy [11], and on the mechanism through which egoistic motivation produces prosocial behavior, such as reciprocity [7], costly signaling [6], and cultural adaptation [4]. These perspectives do not appear helpful to any discussion of the current findings; the first simply posits that the majority of these ‘egoistic’ factors are deceptions, and the second has little interest in the variety among factors.

Given that pro-survival factors are likely to simultaneously benefit the individual and their community, future research should address the social and intrapsychic processes involved in the shared benefit. The social perspective is primarily concerned with how an individual behavior benefits both the individual and community. Shared benefits may be conceptualized by the community-psychology term “social capital” [59] or “socio-psychological resources” [60]. Further research on the different effects of leadership and etiquette on social support will contribute to a deeper understanding of how social capital or resources may be increased in a community or mobilized by individuals. Shared benefits may also be explained in terms of social adaptation, as exemplified by the effects of problem-solving and active well-being on social support. A future research target is further exploration of the social context of this association [25, 27]. Intrapsychic processes concern the psychological and neural processes that produce shared benefit. Within this domain, we only have substantial knowledge regarding the altruism factor; the cascade of processes is assumed to start from the perception of another’s distress, which provokes an empathic response that ultimately drives prosocial behavior [61]. While the processes mediating associations with other power to live factors are poorly understood, some contextual effects on the associations [25, 27] may contribute to hypothesis building. Further advances in understanding could facilitate socio-educational interventions to enhance both individual- and community-level resilience; currently, interventions are largely at the theoretical stage, or are attempted only in certain domains [8, 9].

The present survey was conducted nearly 3 years after the earthquake and tsunami. Thus, any limitations inherent to retrospective surveys, including the effects of biases and limited accuracy in terms of memory, would also apply to this study. There were also theoretical and technical limitations regarding the assessments of representativeness of the present sample. This sample population theoretically included individuals who could potentially provide or receive social support. However, because the potential for such behavior is subjective in nature, it was difficult to define objectively and characterize the population accurately. Furthermore, the present analyses did not include various psychological factors associated with tsunami evacuation [47] that might have affected behavior during the evacuation as well as the subsequent provision and receipt of social support. These factors were not included in the present analyses due to the absence of a specific hypothesis; exploratory analyses that included the interactions of all these factors with personality characteristics would have required unrealistically stringent statistical thresholds for the correction of multiple comparisons. Finally, self-evaluation of one’s own personality may be biased by the retrieval of the experience of social support and being supported. Indeed, personality itself may be affected by the experience of the disaster [62]. Given the difficulties with the prospective approach to this issue, these shortcomings may be best addressed by investigating social and intrapsychic processes in virtual or experimental settings.

## Conclusion

Among the eight pro-survival power to live personality factors, six factors were associated with some type of social support during the emergency evacuation from a tsunami area. Altruism, problem-solving, etiquette, and self-transcendence contributed to actual helping, leadership and active well-being were associated with the provision of oral encouragement, and active well-being was associated with perceived support. The present findings were largely consistent with the reported associations between similar personality factors and social support in non-emergency contexts. Interestingly, none of the Big Five dimensions was associated with social support. Collectively, the high relevance of pro-survival personality factors to social support is congruent with the view that the propensity to cooperate in service of human survival is primarily social, in opposition to the currently dominant view that it is primarily an individualistic phenomenon, as represented by the altruism-egoism dichotomy and evolutionary theories of altruism. Further understanding of the social and intrapsychic processes underlying the joint benefits to the individual and community conferred by these personality factors should make a practical contribution to individual- and community-level resilience.

## Supporting information

S1 Raw data Raw data set. (XLSX)

S1 File Comparisons of demographic information and damage characteristics between the evacuated and non-evacuated respondents. (DOCX)
