# Supplementary material for: Survival-oriented personality factors are associated with various types of social support in an emergency disaster situation
Source: PLoS One. 2020 Feb 12;15(2):e0228875. doi: 10.1371/journal.pone.0228875 (PMC7015700; doi:10.1371/journal.pone.0228875)
Supplement: S1 File — (DOCX) [file pone.0228875.s002.docx]

**Survival-oriented personality factors are associated with various types of social support in an emergency disaster situation**

Motoaki Sugiura^1,2*^, Rui Nouchi^2^, Akio Honda^3^, Shosuke Sato^1^,

Tsuneyuki Abe^4^, Fumihiko Imamura^1^

*^1^ International Research Institute of Disaster Science, Tohoku University, Sendai, Japan*

*^2^ Institute of Development, Aging and Cancer, Tohoku University, Sendai, Japan*

*^3^* *Faculty of Informatics, Shizuoka Institute of Science and Technology, Fukuroi, Japan*

*^4^ Graduate School of Arts and Letters, Tohoku University, Sendai, Japan*

*** Corresponding author**

E-mail: sugiura@tohoku.ac.jp (MS)

**Supplementary data**

Comparison of the evacuated and non-evacuated respondents

For reference purposes regarding data representativeness, the demographic factors (i.e., sex [male or female] and age [20–29, 30–39, 40–49, 50–59, 60–69, or 70+ years]) and damage characteristics (i.e., personal injury [yes or no], home damage [total, partial, or none], and family member loss [yes or no]) of the 959 evacuated and 428 non-evacuated respondents were compared. The data were cross-tabulated, Chi-square tests were performed, and p values < 0.05 (uncorrected) were considered to indicate statistical significance (Table S1).

There was a significant group difference in terms of home damage and, thus, the percentages of effective responses between the two groups were separately compared for the three levels of home damage (Fig. S1). The evacuated respondents suffered from more total home damage than the non-evacuated respondents, which seems to suggest that the different behaviors (i.e., evacuated or non-evacuated) stemmed from the perceived likeliness of a tsunami hit, which was likely to be due to geographic factors (e.g., distance from seacoast). Although it appears unusual that 40% of the non-evacuated respondents experienced total home damage but managed to avoid injury on the whole, this may have been due to the fact that they were not at home when the earthquake occurred in the afternoon (14:46) on 11 March, 2011.

**Table S1 Comparison of the evacuated and non-evacuated responders**

|  |  | **Evacuated** | **Non-evacuated** |  |  |  |  |
| --- | --- | --- | --- | --- | --- | --- | --- |
|  | *N* | 959 | 428 | χ^2^ |  | *df* | Cramer’s *V* |
| **Sex** |  |  |  | 1.178 |  | 1 | 0.029 |
| Male | 556 | 376 | 180 |  |  |  |  |
| Female | 817 | 575 | 242 |  |  |  |  |
| **Age (y)** |  |  |  | 9.009 |  | 6 | 0.081 |
| 20–29 | 13 | 7 | 6 |  |  |  |  |
| 30–39 | 86 | 50 | 36 |  |  |  |  |
| 40–49 | 142 | 95 | 47 |  |  |  |  |
| 50–59 | 213 | 153 | 60 |  |  |  |  |
| 60–69 | 268 | 183 | 85 |  |  |  |  |
| 70+ | 388 | 270 | 118 |  |  |  |  |
| **Personal injury** |  |  |  | 0.238 |  | 1 | 0.013 |
| Yes | 109 | 73 | 36 |  |  |  |  |
| No | 1264 | 875 | 389 |  |  |  |  |
| **Home damage** |  |  |  | 17.966 | * | 2 | 0.114 |
| Total | 672 | 495 | 177 |  |  |  |  |
| Partial | 570 | 379 | 191 |  |  |  |  |
| None | 134 | 76 | 58 |  |  |  |  |
| **Family member loss** |  |  |  | 1.014 |  | 1 | 0.027 |
| Yes | 118 | 86 | 32 |  |  |  |  |
| No | 1224 | 837 | 387 |  |  |  |  |

All data are cross-tabulated with demographic factors (i.e., sex and age) and damage characteristics (i.e., personal injury, home damage, and family member loss). Samples with missing data relevant to the analysis were excluded. Chi-square tests were performed. *p < 0.05, uncorrected. Cramer’s *V* is used as the effect size.

**Figure S1.** Percentages of effective responses between the two groups were separately compared in terms of the three levels of home damage. The significant group difference appears to have stemmed from the occurrence of more total damage in the evacuated respondents group.
